# Supplementary material for: The early human interferon gamma response to Toxoplasma gondii is driven by Vγ9Vδ2 T-cell sensing of host phosphoantigens and subsequent NK-cell activation
Source: PLoS Pathog. 2025 Dec 26;21(12):e1013829. doi: 10.1371/journal.ppat.1013829 (PMC12755758; doi:10.1371/journal.ppat.1013829)
Supplement: S1 Table — RNA-seq data from the GEO dataset GSE119835 were analyzed using GEO2R to compare gene expression between RH strain-infected and media-treated human PBMCs. In short, five PBMCs samples were split into three conditions: untreated, infected with RH88 (MOI 3), or infected with PRU (MOI 3). After 12 hours, RNA was extracted and sequenced (41). Differential expression was assessed by comparing RH-infected to untreated samples. Genes were selected based on known roles in the mevalonate/isoprenoid biosynthesis pathway, host phosphoantigen recognition, or lipid transport. Log2 fold change (log2FC) values represent expression differences (positive = upregulated; negative = downregulated). Adjusted p-values (Benjamini-Hochberg FDR) reflect the statistical significance of differentially expressed genes. (DOCX) [file ppat.1013829.s010.docx]

**Table S1**

| **Gene Symbol** | **Gene Name** | **log2Fold Change** | **Adjusted p-value** | **Pathway / Function** |
| --- | --- | --- | --- | --- |
| SQLE | squalene epoxidase | 1.6 | 4.3e-5 | Cholesterol biosynthesis |
| HMGCS1 | 3-hydroxy-3-methylglutaryl-CoA synthase 1 | 1.3 | 1.7e-5 | Upstream mevalonate synthesis |
| MVK | mevalonate kinase | 1.2 | 2.0e-3 | Mevalonate phosphorylation |
| HMGCR | 3-hydroxy-3-methylglutaryl-CoA reductase | 1.1 | 3.4e-3 | Cholesterol biosynthesis |
| FDPS | farnesyl diphosphate synthase | 0.94 | 1.5e-4 | Isoprenoid synthesis |
| IDI1 | isopentenyl-diphosphate delta isomerase 1 | 1.0 | 1.6e-2 | IPP/DMAPP isomerization |
| MVD | mevalonate diphosphate decarboxylase | 0.95 | 1.7e-3 | Isoprenoid synthesis |
| PMVK | phosphomevalonate kinase | 0.15 | 6.9e-1 | Mevalonate phosphorylation |
| PPP2CA | protein phosphatase 2 catalytic subunit alpha | 0.48 | 1.9e-1 | PP2A catalytic subunit |
| ABCA1 | ATP binding cassette subfamily A member 1 | 0.075 | 9.2e-1 | Cholesterol efflux |
| IFNG | interferon gamma | 7.5 | 2.1e-26 | Cytokine |
| TNF | tumor necrosis factor | 1.7 | 5.1e-3 | Pro-inflammatory cytokine |
| IL12A | interleukin 12A | 0.92 | 7.0e-2 | Cytokine (IL-12 subunit) |
| IL1B | interleukin 1 beta | 3.8 | 2.1e-17 | Pro-inflammatory cytokine |
| BTN3A1 | butyrophilin subfamily 3 member A1 | -0.79 | 1.3e-1 | Phosphoantigen recognition (Vγ9Vδ2 T cell) |
| BTN2A1 | butyrophilin subfamily 2 member A1 | -0.42 | 3.0e-1 | BTN2 family |
| IL18 | interleukin 18 | -0.62 | 4.8e-1 | Inflammasome-related cytokine |
| GGPS1 | geranylgeranyl diphosphate synthase 1 | -0.55 | 1.5e-2 | Isoprenoid synthesis |
