# Supplementary material for: The early human interferon gamma response to Toxoplasma gondii is driven by Vγ9Vδ2 T-cell sensing of host phosphoantigens and subsequent NK-cell activation
Source: PLoS Pathog. 2025 Dec 26;21(12):e1013829. doi: 10.1371/journal.ppat.1013829 (PMC12755758; doi:10.1371/journal.ppat.1013829)
Supplement: S2 Table — (DOCX) [file ppat.1013829.s011.docx]

| **Primer Name** | **Sequence (5' to 3')** | **Gene/Plasmid** | **Use** | **Size** | **Citation** |
| --- | --- | --- | --- | --- | --- |
| GPO-3 FW | GGGAGCAAACAGGATTAGATACCCT | Mycoplasma 16S rRNA | Forward primer to amplify Mycoplasma contamination in cell culture via PCR | 280 bp long will also see non-specific band at 100 bp | Kuppeveld et al. 1994 Applied and Environ. Microbio. 60: 149-152 (51) |
| MGSO RV | TGCACCATCTGTCACTCTGTTAACCTC | Mycoplasma 16s rRNA | Reverse primer to amplify Mycoplasma contamination in cell culture via PCR | 280 bp long will also see non-specific band at 100 bp | Kuppeveld et al. 1994 Applied and Environ. Microbio. 60: 149-152 (51) |
| GRA6 FW | ATTTGTGTTTCCGAGCAGGT | GRA6 | for genotyping at GRA6 locus types I, II, or III, PCR then cut with MseI |  | Made in Saeij Lab |
| GRA6 RV | TCGCCGAAGAGTTGACATAG | GRA6 | for genotyping at GRA6 locus types I, II, or III, PCR then cut with MseI |  | Made in Saeij Lab |
| GlpT FW1 | ATGTTGAGTATTTTTAAACCAGCG | Glycerol 3-transporter | Glycerol 3-transporter confirmation in GlpT and R45K-R269K/ Sequencing | Used for sequencing/ 1360 bp long when using GlpT FW1 and GlpT RV1 | Made in Saeij Lab |
| GlpT RV1 | TTAGCCTCCGTTGCGTTCTT | Glycerol 3-transporter | Glycerol 3-transporter confirmation in GlpT and R45K-R269K | 1360 bp long when using GlpT FW1 and GlpT RV1 | Made in Saeij Lab |
| GlpT FW2 | CGCTCTATATGCCTGCTTTCT | Glycerol 3-transporter | Glycerol 3-transporter confirmation in GlpT and R45K-R269K/ Sequencing | Used for sequencing/ 794 bp when using GlpT FW2 and GlpT RV1 | Made in Saeij Lab |

**Table S2**
